# Supplementary material for: The Construction and Exploration of a Comprehensive MicroRNA Centered Regulatory Network in Foxtail Millet (Setaria italica L.)
Source: Front Plant Sci. 2022 May 6;13:848474. doi: 10.3389/fpls.2022.848474 (PMC9121102; doi:10.3389/fpls.2022.848474)
Supplement: Supplementary file 6 [file Data_Sheet_6.zip › Sit-miRN2531_Seita.9G120600.1_1389_TPlot.pdf]

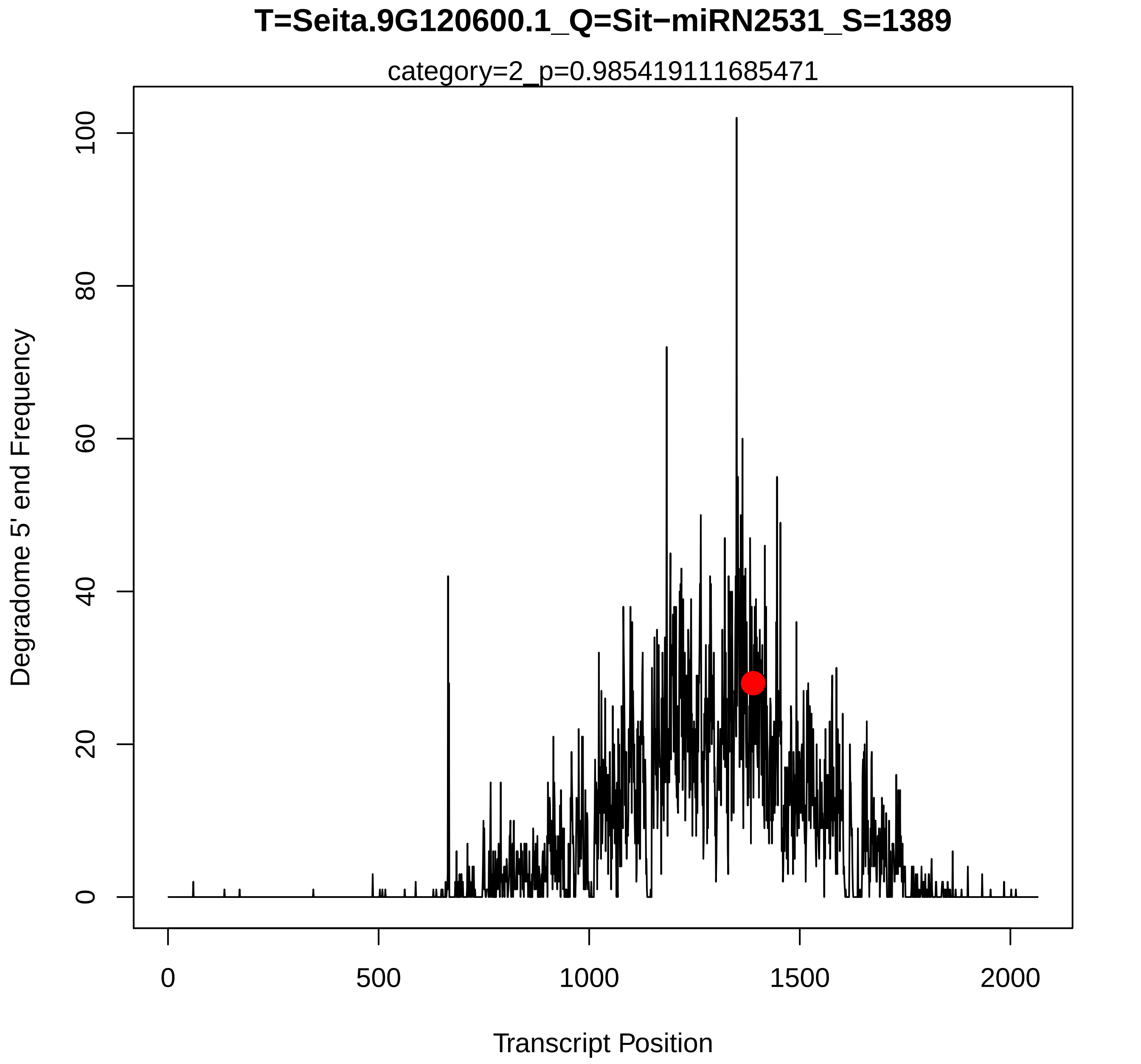

T=Seita.9G120600.1_Q=Sit−miRN2531_S=1389
category=2_p=0.985419111685471
Degradome 5' end Frequency
Transcript Position
0
20
40
60
80
100
0
500
1000
1500
2000
